# Pretreatment vitamin D level and response to neoadjuvant chemotherapy in women with breast cancer on the I-SPY trial (CALGB 150007/150015/ACRIN6657)

**DOI:** 10.1002/cam4.235

**Published:** 2014-04-09

**Authors:** Amy S Clark, Jinbo Chen, Shiv Kapoor, Claire Friedman, Carolyn Mies, Laura Esserman, Angela DeMichele, for the I-SPY1 Investigators

**Affiliations:** 1 Abramson Cancer CenterPhiladelphia, Pennsylvania; 2 Perelman School of Medicine at the University of PennsylvaniaPhiladelphia, Pennsylvania; 3 Division of Hematology/Oncology, Perelman School of Medicine at the University of PennsylvaniaPhiladelphia, Pennsylvania; 4 Center for Clinical Epidemiology and Biostatistics, Perelman School of Medicine at the University of PennsylvaniaPhiladelphia, Pennsylvania; 5 Hospital of the University of PennsylvaniaPhiladelphia, Pennsylvania; 6 Department of Pathology, Perelman School of Medicine at the University of PennsylvaniaPhiladelphia, Pennsylvania; 7 Helen Diller Family Comprehensive Cancer CenterSan Francisco, California; 8 UCSF/Mount Zion Medical CenterSan Francisco, California

**Keywords:** Breast cancer, neoadjuvant chemotherapy, response, vitamin D

## Abstract

Laboratory studies suggest that vitamin D (vitD) enhances chemotherapy-induced cell death. The objective of this study was to determine whether pretreatment vitD levels were associated with response to neoadjuvant chemotherapy (NACT) in women with breast cancer. Study patients (*n* = 82) were enrolled on the I-SPY TRIAL, had HER2-negative tumors, and available pretreatment serum. VitD levels were measured via DiaSorin radioimmunoassay. The primary outcome was pathologic residual cancer burden (RCB; dichotomized 0/1 vs. 2/3). Secondary outcomes included biomarkers of proliferation, differentiation, and apoptosis (Ki67, grade, Bcl2, respectively) and 3-year relapse-free survival (RFS). Mean and median vitD values were 22.7 ng/mL (SD 11.9) and 23.1 ng/mL, respectively; 72% of patients had levels deemed “insufficient” (<30 ng/mL) by the Institute of Medicine (IOM). VitD level was not associated with attaining RCB 0/1 after NACT (univariate odds ratio [OR], 1.01; 95% CI, 0.96–1.05) even after adjustment for hormone receptor status (HR), grade, Ki67, or body mass index (BMI). Lower vitD levels were associated with higher tumor Ki67 adjusting for race (OR, 0.95; 95% CI, 0.90–0.99). VitD level was not associated with 3-year RFS, either alone (hazard ratio [HzR], 0.98; 95% CI, 0.95–1.02) or after adjustment for HR, grade, Ki-67, BMI, or response. VitD insufficiency was common at the time of breast cancer diagnosis among women who were candidates for NACT and was associated with a more proliferative phenotype. However, vitD levels had no impact on tumor response to NACT or short-term prognosis.

## Introduction

Approximately 230,000 new cases of breast cancer were diagnosed in 2013; 95% of these cases are theoretically curable 1. Those at the highest risk of distant relapse have large primary tumors, lymph node involvement, or estrogen, progesterone, and HER2-negative (TN) disease 2. Achievement of pathologic complete response following neoadjuvant chemotherapy (NACT) predicts for improved survival 3–6 in this high-risk population, particularly in those with TN or HER2+ breast cancer as demonstrated in the I-SPY1 and other trials 7–10. Despite its potential high curability, 39,620 women died from breast cancer in 2013 11. Therefore, modifiable factors that improve therapeutic efficacy and decrease relapse continue to be sought.

Vitamin D (vitD) deficiency (defined by the Institute of Medicine [IOM] as <20 ng/mL 12) is common, occurring in ∼40% of the general adult U.S. population 13. VitD maintains calcium homeostasis and bone metabolism, and may play a role in malignancy. Ecological studies demonstrate higher breast cancer deaths in areas with less sunlight 14. Moreover, scientific evidence suggests that calcitriol, the steroid active metabolite of vitD, is important for regulation of the cell cycle. Through binding to vitD receptors (VDRS), ubiquitously expressed in epithelial cells, including those in the normal and malignant breast cells 15, calcitriol directly and indirectly influences transcription, resulting in inhibition of breast cancer cellular proliferation while inducing differentiation and apoptosis 15. In vitro and in vivo data further demonstrate that calcitriol augments chemotherapy-induced cell death 16–22. This has not been explored in breast cancer patients to our knowledge.

Because of the intracellular effects on breast cancer cells and the in vitro and in vivo experiments demonstrating enhancement of chemotherapeutic cytotoxicity with vitD pretreatment, we hypothesized that low vitD levels would be associated with impaired response to chemotherapy and more aggressive breast tumor biology resulting in higher relapse rates among women with vitD deficiency. To address these hypotheses, we measured pretreatment vitD levels in a cohort of women with newly diagnosed breast cancer enrolled on the I-SPY1 Trial (CALGB 150007/150015/ACRIN6657). Our primary aim was to determine the relationship between vitD levels and response to NACT. Our secondary aims examined the relationship between vitD levels and biomarkers of proliferation, cell death, and differentiation as well as breast cancer relapse-free survival (RFS).

## Methods

We performed a retrospective cohort study examining pretreatment vitD levels in frozen serum obtained at enrollment in patients on the I-SPY1 Trial. As previously described 7, I-SPY1 was a multicenter, prospective cohort study that examined biomarkers and radiographic predictors of response to NACT. Serial breast biopsies and breast magnetic resonance imaging (MRI) scans were obtained before and during chemotherapy treatment. All subjects enrolled on I-SPY1 had pretreatment serum samples drawn, processed, and stored at the CALGB pathology core facility.

To be included in our study, subjects had to have provided written, informed consent to the parent trial and have frozen serum available for vitD testing. The eligibility criteria for I-SPY1 are described in detail elsewhere 8. Briefly, patients had histologically confirmed breast cancer that was 3.0 cm or greater without evidence of distant metastatic disease. All patients received NACT with an anthracycline, 90% also received a taxane, and 98% ultimately underwent definitive surgery. Women with HER2-overexpressing tumors were excluded from our study because serum samples were not available.

Serum samples collected at enrollment were stored in aliquots at −80°C. 25(OH)D levels were measured using the DiaSorin radioimmunoassay as previously described (DiaSorin, Stillwater, MN) in the Clinical Research Center of the Perelman School of Medicine at the University of Pennsylvania 23,24. Data were analyzed using vitD as a continuous and dichotomous variable. Cut points for the dichotomous variables were based on the IOM definitions of vitD deficiency and insufficiency (<20 vs. ≥20 ng/mL; <30 vs. ≥30 ng/mL, respectively).

### Outcome measurements

Our primary outcome was response to NACT and was dichotomized into response and no response. We defined response as an residual cancer burden (RCB) of 0 or 1 (complete or near-complete resolution of invasive breast cancer in the breast and lymph nodes as defined by Symmans et al. 25). No response was defined as RCB of 2 or 3 (stable or progression of disease).

Secondary outcomes were tumor expression of Ki67, Bcl2, and tumor grade and RFS. Ki67, Bcl2, and tumor grade were measured from the original tumor biopsy specimens by one pathologist at the University of North Carolina, as previously described 26. Ki67 and Bcl2 were dichotomized <10% and ≥10% 27,28. Low grade was compared with moderate and high grade. Three-year RFS was collected by I-SPY1. RFS was defined according to Standardization of Events and End Points criteria 29 and began on the first day of chemotherapy. All covariable data were obtained from the I-SPY1 database; missing weight data were obtained if available from clinical sites directly.

### Statistical analysis

Patient characteristics from this study were compared to the remainder of the HER2 negative I-SPY1 study population using *χ*^2^ analysis. We assessed vitD level distribution by generating histograms and examined the mean and median vitD levels. We compared median vitD levels in different population subgroups defined by covariables using the Wilcoxon rank-sum test or the Wilcoxon rank-sum test for trend when a covariable had more than two groups. We used logistic regression to examine the relationships between the covariables comparing the RCB 0/1 group with the RCB 2/3 group. We also used logistic regression to examine the relationship between vitD and response. For secondary aims, logistic regression was used to examine the relationship between vitD and measures of tumor biology. Cox proportional regression analysis was performed to assess the association between vitD and RFS. Kaplan–Meier curves were created to display the experience of RFS for patients with vitD bivariable levels. We built models to adjust individually for potential confounding covariates and performed analyses to explore potential vitD and covariate interaction. Multivariate modeling was restricted to one covariate per model (trivariate models) due to the limitation in our sample size. Subjects with missing variables were excluded from analyses involving the specific variable. All statistical analyses were performed using STATA version 12.0 (College Station, TX).

For power calculations, we estimated that the mean vitD level would be 20 ng/mL with a standard deviation of 9 based on a recent NHANES study 13. From a fixed sample size of 82 subjects with 17 responders (RCB 0/1), our study had 80% power to detect a change in odds of response of 9.5% for every 1 ng/mL change in vitD level with a two-sided test of significance level = 0.05.

## Results

Of the 221 subjects enrolled on I-SPY1, 64 were excluded from the current analysis due to HER2 positivity and 75 were excluded due to lack of remaining frozen serum. In total, 83 serum samples from 82 subjects were analyzed. Compared to those without samples, patients in the vitD cohort were more likely to be from the northern United States (*P* = 0.005, Table 1). Otherwise, excluded subjects did not differ from study subjects (Table 1). Pathologic response in the vitD cohort was significantly associated with HR negative (OR, 0.26; 95% CI, 0.15–0.47), moderate or high tumor grade (OR, 9.64; 95% CI, 1.26–73.93), low levels of Bcl2 (OR, 0.41; 95% CI, 0.22–0.79), and Ki67 >10 (OR, 2.46; 95% CI, 1.13–5.31).

**Table 1:** Patient characteristics

	I-SPY (*n* = 221)	Vitamin D (*n* = 82)	Non-vitamin D^1^ (*n* = 75)	*P*-value^1^
Mean age, years (SD)	48.3 (8.9)	48.1 (9.0)	48.0 (9.5)	0.94
Race, *n* (%)				
Caucasian	165 (75%)	61 (75%)	59 (79%)	
Non-Caucasian	54 (25%)	20 (25%)	15 (20%)	0.51
BMI, *n* (%)				
<25 kg/m^2^	66 (30%)	29 (35%)	20 (27%)	
25–30 kg/m^2^	45 (20%)	18 (22%)	15 (20%)	
>30 kg/m^2^	57 (26%)	31 (38%)	13 (17%)	0.32
Location, *n* (%)				
North	131 (59%)	40 (49%)	53 (71%)	
South	90 (41%)	42 (51%)	22 (29%)	0.005
Hormone receptor, *n* (%)				
Positive	131 (59%)	50 (61%)	50 (67%)	
Negative	90 (41%)	32 (39%)	25 (33%)	0.46
Grade, *n* (%)				
Low	18 (8%)	8 (10%)	9 (9%)	
Intermediate	96 (44%)	30 (37%)	38 (51%)	
High	103 (47%)	42 (51%)	27 (36%)	0.13
Chemotherapy *n* (%)				
AC, T or equivalent	208 (94%)	75 (91%)	71 (95%)	
Other	13 (6%)	7 (9%)	4 (5%)	0.43
Response *n* (%)				
pCR	58 (26%)	12 (15%)	17 (23%)	
No pCR	157 (71%)	70 (85%)	58 (77%)	0.20
Residual cancer burden, *n* (%)				
RCB 0	58 (26%)	12 (15%)	17 (23%)	
RCB 1	18 (8%)	5 (6%)	4 (5%)	
RCB 2	86 (39%)	45 (55%)	24 (32%)	
RCB 3	41 (19%)	15 (16%)	15 (20%)	0.08
Outcomes, *n* (%)				
Alive	176 (80%)	64 (78%)	59 (78%)	0.56
Relapse-free	160 (72%)	59 (72%)	57 (76%)	0.28

*P* value calculated based on *χ*^2^ analysis comparing the vitD to non-vitD HER2 negative I-SPY1 subgroups. BMI, body mass index; AC, T, doxorubicin, cyclophosphamide, paclitaxel; pCR, pathologic complete response; RCB, residual cancer burden.

1 Non-vitamin D group represents the HER2 negative I-SPY patients without remaining serum for analysis.

All 83 serum samples were successfully assayed for 25(OH)D. The intra-assay coefficient of variation was 8.81%. The vitD distribution was right-skewed. The mean and median vitD levels were 22.1 ng/mL (SD 11.9) and 23.4 ng/mL, respectively (Table 2). As per the IOM definitions, 41% had deficient and 72% had insufficient vitD levels. VitD level was associated with race (median level 26.2 ng/mL in Caucasians vs. 11.5 ng/mL in non-Caucasians; *P* = 0.0001), higher body mass index (BMI) (*P*-trend=0.01 comparing those <25 kg/m^2^ to those 25–30 kg/m^2^ to those >30 kg/m^2^), season of blood draw (median 17.5 ng/mL if drawn during winter or spring compared to 27.3 ng/mL summer or fall, *P* = 0.009) and geographic location of blood draw (median level from subjects from northern cities was 25.8 and 18.3 ng/mL in those from southern cities, *P* = 0.011). Notably, the vitD/geographic association was the reverse of expected because 80% of subjects from southern cities were of non-Caucasian race (*P* = 0.002, data not shown); thus, the high proportion of non-Caucasian subjects in southern locations accounted for this inverse association. VitD levels were not associated with HR.

**Table 2:** Vitamin D levels among different study subgroups

	*N*	Mean (SD), ng/mL	Median, ng/mL	Interquartile range, ng/mL	*P*-value^1^
Total	82	22.7 (11.9)	23.1	13.1, 30.5	N/A
Age (years)					
<45	25	26.7 (15.1)	26.8	15.3, 32.9	0.14 (trend)
45–55	39	22.1 (9.7)	22.6	12.9, 30.3	
>55	18	20.3 (10.4)	19.3	12.0, 27.4	
Race					
Caucasian	61	25.9 (11.7)	26.2	16.0, 32.5	0.0001
Non-Caucasian	20	14.4 (8.1)	11.5	8.5, 19.4	
BMI (kg/m^2^)					
<25	29	27.0 (11.5)	27.3	18.8, 32.5	0.01 (trend)
25–30	18	19.7 (7.7)	20.2	13.6, 25.6	
>30	31	19.8 (12.1)	14.4	9.1, 31.2	
Location					
North	40	26.4 (11.6)	25.8	17.9, 32.5	0.011
South	42	20.0 (11.5)	18.3	9.2, 28.5	
Season of blood draw					
Winter/spring	31	19.6 (9.4)	17.5	12.4, 30.5	0.009
Summer/fall	45	27.2 (12/4)	27.3	20.0, 32.6	
Hormone receptor					
Positive	50	24.4 (12.0)	24.9	11.3, 29.5	0.21
Negative	31	21.0 (11.6)	20.7	14.2, 31.4	
Response					
pCR	12	25.3 (8.7)	26.8	16.0, 31.2	0.44
no pCR	69	22.6 (12.4)	22.7	12.9, 30.4	
Residual cancer burden					
RCB 0/1	17	23.9 (9.9)	26.8	16.0, 31.2	0.34 (trend)
RCB 2	45	23.5 (12.7)	22.7	13.1, 30.5	
RCB 3	15	21.6 (12.6)	21.4	12.0, 27.1	

BMI, body mass index; pCR, pathologic complete response; RCB, residual cancer burden.

1 Comparison of median vitamin D values (across strata when testing for trend) using Wilcoxon rank-sum test or Wilcoxon rRank-sum test for trend.

The median vitD level in those achieving a response (RCB 0/1) to NACT was 26.8 ng/mL compared to 21.9 ng/mL in those without a response (RCB 2/3) (Table 2). Univariate analysis did not demonstrate a statistically significant association between vitD levels and response to NACT (OR, 1.01; 95% CI, 0.96–1.05) (Table 3). Individual adjustment by potential patient-related confounders (race and BMI) or tumor and response-related confounders (HR, Ki67, and grade), did not alter the association. When stratified by HR or Ki67, there was no evidence of effect modification (*P* interaction 0.43 for HR and 0.95 for Ki67).

**Table 3:** Vitamin D and response: examination in three models

Model (*N*)	Vitamin D (continuous) OR (95% CI) *P*-value	Vitamin D deficiency OR (95% CI) *P*-value	Vitamin D insufficiency OR (95% CI) *P*-value
Univariate analysis		≥20 vs. <20 ng/mL	≥30 vs. <30 ng/mL
Vitamin D (82)	1.01 (0.96, 1.05) *P* = 0.391	0.75 (0.14, 2.19) *P* = 0.535	1.54 (0.49, 4.80) *P* = 0.253
Multivariate analysis			
Race (81)	1.01 (0.97, 1.07) *P* = 0.323	0.83 (0.26, 2.68) *P* = 0.408	1.68 (0.51, 5.53) *P* = 0.271
HR (82)	1.02 (0.97, 1.07) *P* = 0.571	0.83 (0.27, 2.54) *P* = 0.708	1.75 (0.53, 5.81) *P* = 0.263
Grade (80)	1.01 (0.96, 1.06) *P* = 0.443	0.82 (0.26, 2.62) *P* = 0.527	1.66 (0.47, 5.84) *P* = 0.312
Ki67 (73)	1.02 (0.97, 1.07) *P* = 0.506	0.98 (0.30, 3.15) *P* = 0.606	1.78 (0.50, 6.30) *P* = 0.452
BMI (78)	1.00 (0.96, 1.05) *P* = 0.133	0.69 (0.22, 2.13) *P* = 0.219	1.37 (0.41, 4.55) *P* = 0.166
Stratified analysis			
HR+^1^ (50)	1.00 (0.93, 1.07) *P* = 0.908	0.26 (0.04, 1.58) *P* = 0.915	1.19 (0.19, 7.33) *P* = 0.574
HR−1 (32)	1.03 (0.97, 1.10) *P* = 0.468	1.93 (0.43, 8.61) *P* = 0.646	2.43 (0.47, 12.54) *P* = 0.367
Ki67 Low^1^ (22)	1.01 (0.93, 1.10) *P* = 0.932	0.71 (0.05, 9.70) *P* = 0.517	4.33 (0.33, 57.65) *P* = 0.821
Ki67 High^1^ (51)	1.02 (0.96, 1.08) *P* = 0.373	1.05 (0.29, 3.84) *P* = 0.354	1.29 (0.28, 5.91) *P* = 0.460

OR, odds ratio; CI, confidence interval; HR, hormone receptor status; BMI, body mass index.

1 There is no evidence of effect modification by HR or Ki67, *P* interaction = 0.43 for HR and 0.95 for Ki67 in continuous model.

**Figure 1:**
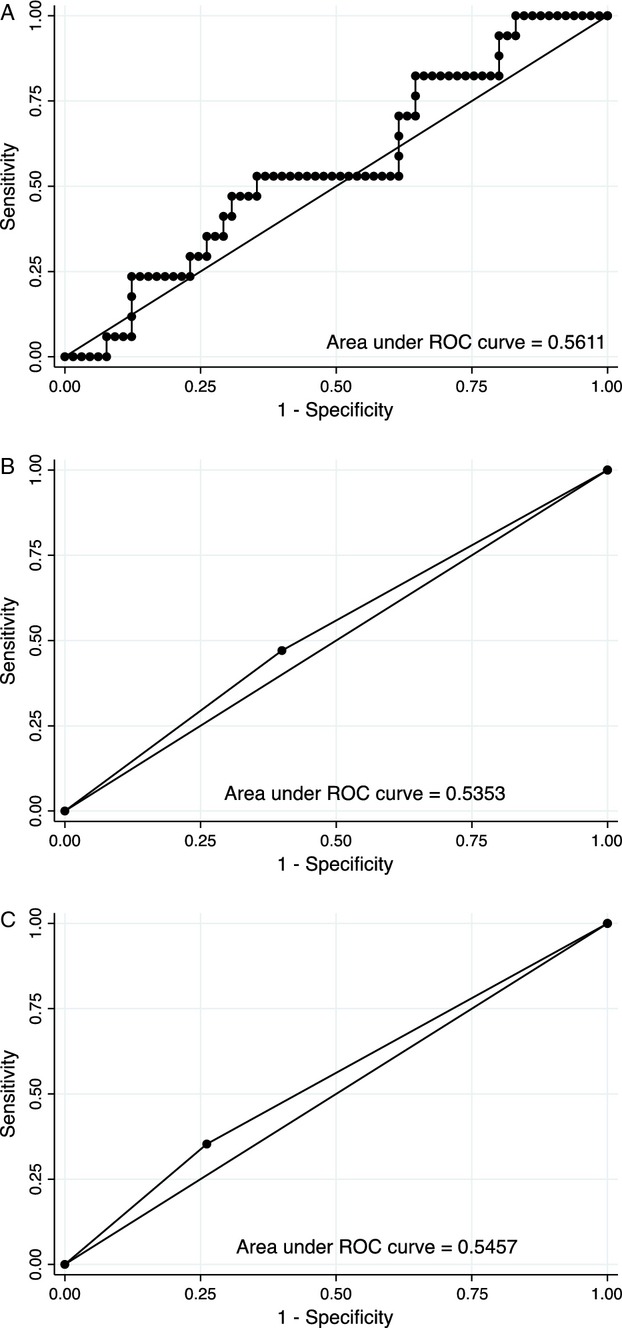
ROC curves: vitamin D levels predicting response to neoadjuvant chemotherapy. (A) ROC to predict response using vitD as a continuous variable. (B) ROC to predict response in those with vitD levels ≥20 or <20 ng/mL. (C) ROC to predict response in those with vitD levels ≥30 or <30 ng/mL.

We further explored the vitD/response relationship by dichotomizing vitD based on the IOM definitions of vitD deficiency and insufficiency. We found no evidence of a significant association between response to NACT and vitD deficiency (OR, 0.75; 95% CI, 0.14–2.19). VitD sufficiency was associated with increased odds of response compared to those with insufficient vitD levels, although this was not statistically significant (OR, 1.54; 95% CI, 0.49–4.80) (Table 3). Receiver operating characteristic curve (ROC) analysis showed that vitD was not a good predictor of response (Fig. 1).

We also performed exploratory analyses to assess the relationship between vitD and biomarkers of tumor biology, including Ki67, tumor grade, and Bcl2 phosphorylation. We found a significant relationship between Ki67 and vitD level (OR, 0.95; 95% CI, 0.91–0.99; *P* = 0.017; Table 4); for every one unit increase in serum vitD level, the odds of having a Ki67 >10 decreased by 5%. This relationship remained unchanged after adjustment for race, HR, grade, or Bcl2 expression (Table 4). Stratifying by HR or response status revealed that neither variable modified the Ki67/vitD relationship (*P* interaction = 0.20 for HR and 0.51 for response).

**Table 4:** Vitamin D and tumor characteristics

	*N*	OR	95% CI	*P*-value^1^
Univariate analysis				
Ki67	73			
Ki67 ≤10	22	1.00	0.91, 0.99	0.017
Ki67 >10	51	0.95		
Bcl2	71			
Bcl2 low	30	1.00	0.98, 1.06	0.348
Bcl2 high	41	1.02		
Grade	80			
Grade 1	8	1.00	0.93, 1.04	0.612
Grade 2/3	72	0.98		
Multivariate analysis with Ki67				
HR	73	0.95	0.90, 1.00	0.04
Race	73	0.95	0.90, 0.99	0.030
Bcl2	73	0.95	0.90, 1.00	0.036
Grade	73	0.95	0.91, 0.99	0.022
Stratified analysis with Ki67				
Ki67, HR+	45	0.93	0.87, 0.99	0.027
Ki67, HR−	28	1.02	0.89, 1.17	0.755

OR, odds ratio; CI, confidence interval; HR, hormone receptor status.

1 *P* value was calculated based on the standard Wald statistic.

**Figure 2:**
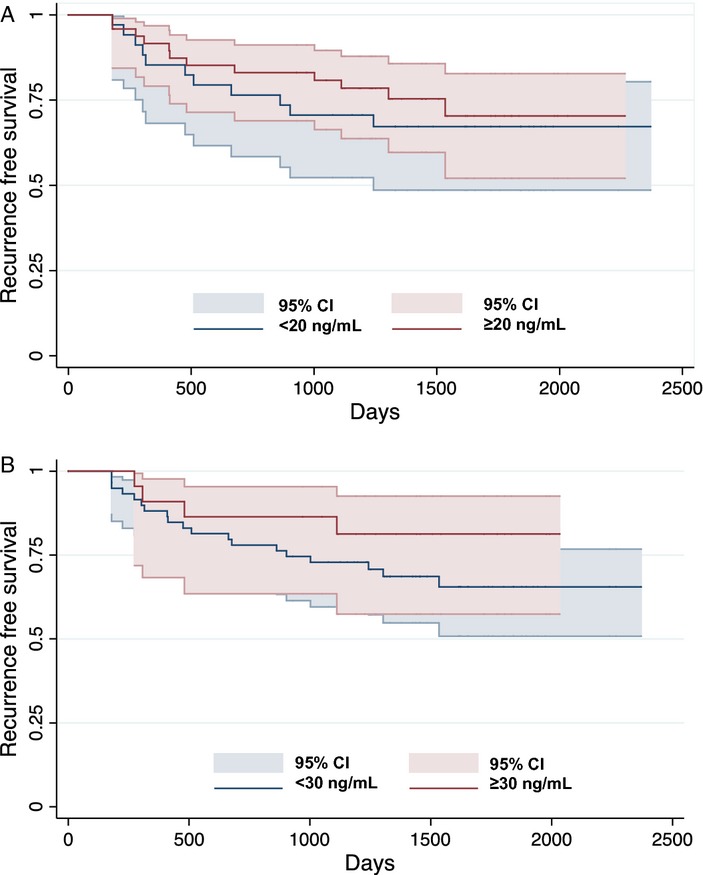
Kaplan–Meier analysis using IOM cutoffs to dichotomize vitamin D. (A) VitD deficiency and RFS (comparing those with levels ≥20 to <20 ng/mL). (B) VitD insufficiency and RFS (comparing those with levels ≥30 to <30 ng/mL).

We did not find a statistically significant relationship between vitD level and breast cancer RFS, with a minimum follow up of 3 years (Table 5). This relationship was not altered after individual adjustment by patient-related (age, race, or BMI) or tumor-related (HR, Ki67, grade, or Bcl2) variables. It was also not altered by stratification on HR, response, or Bcl2 expression (*P* interaction 0.75 for HR, 0.10 for response, and 0.21 for Bcl2). Finally, we explored whether 3-year RFS was related to vitD deficiency and insufficiency. There was no statistically significant relationship in our analysis, although the Kaplan–Meier curves do show some separation between those above and below the deficiency and insufficiency thresholds (Fig. 2).

**Table 5:** Vitamin D and recurrence-free survival

	*N*	HzR	95% CI	*P*-value^1^
Univariate analysis				
Vitamin D, continuous	82	0.98	0.95–1.02	0.391
Deficiency	82	0.77	0.34–1.75	0.535
Insufficiency	82	0.53	0.18–1.57	0.253
Multivariate analysis with continuous vitamin D				
HR	82	0.99	0.95–1.03	0.571
Grade	80	0.99	0.94–1.04	0.778
Response	82	0.99	0.95–1.02	0.441
Age	82	0.98	0.95–1.02	0.383
Race	81	0.97	0.94–1.02	0.323
Chemo	82	0.99	0.95–1.02	0.444
Bcl2	71	0.99	0.95–1.03	0.578
Ki67	73	0.99	0.95–1.03	0.506
BMI	78	0.96	0.92–1.01	0.133
Stratified analysis				
HR+	50	1.00	0.95–1.05	0.908
HR−	32	0.98	0.93–1.03	0.468
RCB 0/1	17	0.99	0.96–1.03	0.583^2^
RCB 2/3	60	0.64	0.26–1.61	0.349^2^
BMI < 25	29	0.99	0.94–1.05	0.828
BMI ≥ 25	49	0.93	0.87–1.01	0.073

HzR, hazard ratio; CI, confidence interval; HR, hormone receptor status; BMI, body mass index; RCB, residual cancer burden.

1 *P* value based on the standard Wald statistic.

2 There is no effect modification by response status (*P* interaction = 0.099).

## Discussion

This study examined the relationship between vitD levels and response to NACT in breast cancer patients. As expected, vitD insufficiency was highly prevalent in our population: 42% were deficient (consistent with general U.S. population statistics) and 72% had insufficient levels per IOM definitions. Significantly lower vitD levels were observed in those of non-Caucasian race, with higher BMI and whose pretreatment serum was drawn in the winter or spring months as expected from previous studies 30–36. Despite adequate power to show a 9.5% change in odds of response for every one unit change in vitD level, we did not find an association between vitD level and response to NACT. In exploratory analyses, we did find a significant inverse association between vitD level and Ki67; for every one unit decrease in vitD level the odds of having a highly proliferative breast tumor (Ki67 >10) increased by 5%. This suggests that higher vitD levels may suppress proliferation of breast tumors, although this is speculative. There was no association between vitD level and RFS.

The rationale for our study was based on scientific evidence demonstrating that calcitriol binds to VDRS in breast cancer cells resulting in direct and indirect inhibition of proliferation through regulation of genes encoding cyclin proteins, cyclin-dependent kinases (CDK), and CDK inhibitors and indirect promotion of differentiation, apoptosis, and angiogenesis in breast cancer cells 15,37–39. When combined in vitro, calcitriol augmented MCF7 breast cancer cell line cytotoxicity via reduction in super oxide dismutase mRNA levels and suppression of Bcl2 protein levels rendering the cells more susceptible to doxorubicin 16 and paclitaxel 19, respectively, compared to either chemotherapeutic alone 16,17,19,40. In vivo models combined calcitriol analogs with doxorubicin, paclitaxel, or tamoxifen and demonstrated enhanced antitumor effect compared these agents alone 18,19,41. Thus, calcitriol appeared to increase chemotherapy-induced cell death in cancer cell lines and animal models, but whether calcitriol influences chemotherapy efficacy in breast cancer patients had not previously been explored.

We acknowledge several limitations to this study. First, our sample size was fixed at 82 subjects with 17 responders in HER2 negative subjects. Thus, we were unable to build full multivariate models, though we did perform trivariate analyses to assess for confounding by individual covariables. When vitD was used as a continuous variable, we did not find evidence for confounding by any of the variables we assessed. In trivariate analysis, the only covariable that significantly contributed to the vitD/response relationship was HR. Although HR was significant in the model, the vitD/response relationship remained insignificant. Thus, we doubt that a full model would change these results. Despite the small sample size, our study was adequately powered to definitively detect whether a relationship exists between vitD levels and response.

Second, we note that the vitD levels in our population were skewed with only 28% having sufficient levels (>30 ng/mL). The OR for univariate and adjusted models comparing subjects with vitD insufficiency to those with sufficient levels were consistently over 1.0 suggesting an association between vitD sufficiency and response. We were not powered to answer this question, and further studies are needed to specifically explore this association. Furthermore, it is scientifically plausible that vitD levels needed to optimize breast health and response to chemotherapy have no relationship with the IOM cutoffs, since these were based on bone events. Supporting this idea is the unadjusted dose-response curve calculated by Mohr et al. in a recent meta analysis that estimated a 50% lower risk of breast cancer in subjects with serum vitD levels above 47 ng/mL compared to those with levels <47 ng/mL 42. If these vitD levels were also needed to optimize response to chemotherapy, our study would not be able to identify such an association since so few subjects had levels above 30 ng/mL.

Third, there are potentially important unmeasured confounding variables, such as smoking status, physical activity, diabetes mellitus type 2 status, or vitD supplement use. None of these were collected as part of the I-SPY study. While these variables have been associated with breast cancer relapse, there has been no defined association between these variables and response to breast cancer chemotherapy which was our primary endpoint. Thus, we doubt that inclusion of any of these covariables would uncover a statistically significant relationship between vitD and response. Finally, we do not know who was taking vitD supplements and at what dose. It is plausible that some might have taken high doses, and this might bias our results toward the null.

We also did not find evidence that vitD levels were of prognostic significance for those with a new breast cancer diagnosis. Our RFS analyses were negative, although few events had occurred, limiting the power of this analysis (post hoc analysis revealed 10% power to detect a difference in relapse between those with vitD levels ≥ and <30 ng/mL). Moreover, Hatse et al. have found a statistically significant difference between vitD and relapse over time 43. We note that the Kaplan–Meier curves for those with vitD levels above and below the insufficient range do separate, favoring those with levels above 30 ng/mL.

Our study suggests that insufficient or deficient vitD levels do not impair or predict the efficacy of NACT in breast cancer patients. We do not know whether the vitD status of our patients was known at the time of NACT. Therefore, our study was not designed to examine whether vitD can enhance the effect of chemotherapy. There is one phase III prospective randomized trial that completed enrollment and compares standard versus high-dose vitD supplementation on time to breast cancer progression. Thus, it still remains to be seen whether vitD supplements can enhance the cytotoxic effects of chemotherapy in breast cancer patients.

## References

[1] Siegel R, Naishadham D, Jemal A (2013). Cancer statistics, 2013. CA Cancer J. Clin.

[2] Foulkes WD, Smith IE, Reis-Filho JS (2010). Triple-negative breast cancer. N. Engl. J. Med.

[3] Wolmark N, Wang J, Mamounas E, Bryant J, Fisher B (2001). Preoperative chemotherapy in patients with operable breast cancer: nine-year results from National Surgical Adjuvant Breast and Bowel Project B-18. J. Natl. Cancer Inst. Monogr.

[4] Machiavelli MR, Romero AO, Perez JE, Lacava JA, Dominguez ME, Rodriguez R (1998). Prognostic significance of pathological response of primary tumor and metastatic axillary lymph nodes after neoadjuvant chemotherapy for locally advanced breast carcinoma. Cancer J. Sci. Am.

[5] Rastogi P, Anderson SJ, Bear HD, Geyer CE, Kahlenberg MS, Robidoux A (2008). Preoperative chemotherapy: updates of National Surgical Adjuvant Breast and Bowel Project Protocols B-18 and B-27. J. Clin. Oncol.

[6] Ferriere JP, Assier I, Cure H, Charrier S, Kwiatkowski F, Achard JL (1998). Primary chemotherapy in breast cancer: correlation between tumor response and patient outcome. Am. J. Clin. Oncol.

[7] Esserman LJ, Berry DA, Demichele A, Carey L, Davis SE, Buxton M (2012). Pathologic complete response predicts recurrence-free survival more effectively by cancer subset: results from the I-SPY 1 TRIAL–CALGB 150007/150012, ACRIN 6657. J. Clin. Oncol.

[8] Esserman LJ, Berry DA, Cheang MC, Yao C, Perou CM, Carey L (2012). Chemotherapy response and recurrence-free survival in neoadjuvant breast cancer depends on biomarker profiles: results from the I-SPY 1 TRIAL (CALGB 150007/150012; ACRIN 6657). Breast Cancer Res. Treat.

[9] Houssami N, Macaskill P, von Minckwitz G, Marinovich ML, Mamounas E (2012). Meta-analysis of the association of breast cancer subtype and pathologic complete response to neoadjuvant chemotherapy. Eur. J. Cancer.

[10] von Minckwitz G (2012). Neoadjuvant chemotherapy in breast cancer-insights from the German experience. Breast Cancer.

[11] Siegel R, Naishadham D, Jemal A (2012). Cancer statistics, 2012. CA Cancer J. Clin.

[12] Ross AC, Manson JE, Abrams SA, Aloia JF, Brannon PM, Clinton SK (2011). The 2011 report on dietary reference intakes for calcium and vitamin D from the Institute of Medicine: what clinicians need to know. J. Clin. Endocrinol. Metab.

[13] Forrest KY, Stuhldreher WL (2011). Prevalence and correlates of vitamin D deficiency in US adults. Nutr. Res.

[14] Robsahm TE, Tretli S, Dahlback A, Moan J (2004). Vitamin D3 from sunlight may improve the prognosis of breast-, colon- and prostate cancer (Norway). Cancer Causes Control.

[15] Deeb KK, Trump DL, Johnson CS (2007). Vitamin D signalling pathways in cancer: potential for anticancer therapeutics. Nat. Rev. Cancer.

[16] Ravid A, Rocker D, Machlenkin A, Rotem C, Hochman A, Kessler-Icekson G (1999). 1,25-Dihydroxyvitamin D3 enhances the susceptibility of breast cancer cells to doxorubicin-induced oxidative damage. Cancer Res.

[17] Wang Q, Yang W, Uytingco MS, Christakos S, Wieder R (2000). 1,25-Dihydroxyvitamin D3 and all-trans-retinoic acid sensitize breast cancer cells to chemotherapy-induced cell death. Cancer Res.

[18] Koshizuka K, Koike M, Asou H, Cho SK, Stephen T, Rude RK (1999). Combined effect of vitamin D3 analogs and paclitaxel on the growth of MCF-7 breast cancer cells in vivo. Breast Cancer Res. Treat.

[19] Koshizuka K, Koike M, Kubota T, Said J, Binderup L, Koeffler HP (1998). Novel vitamin D3 analog (CB1093) when combined with paclitaxel and cisplatin inhibit growth of MCF-7 human breast cancer cells in vivo. Int. J. Oncol.

[20] Hershberger PA, Yu WD, Modzelewski RA, Rueger RM, Johnson CS, Trump DL (2001). Calcitriol (1,25-dihydroxycholecalciferol) enhances paclitaxel antitumor activity in vitro and in vivo and accelerates paclitaxel-induced apoptosis. Clin. Cancer Res.

[21] Ma Y, Yu WD, Trump DL, Johnson CS (2010). 1,25D3 enhances antitumor activity of gemcitabine and cisplatin in human bladder cancer models. Cancer.

[22] Anzano MA, Smith JM, Uskokovic MR, Peer CW, Mullen LT (1994). 1 alpha,25-Dihydroxy-16-ene-23-yne-26,27-hexafluorocholecalciferol (Ro24-5531), a new deltanoid (vitamin D analogue) for prevention of breast cancer in the rat. Cancer Res.

[23] Hollis BW (1997). Quantitation of 25-hydroxyvitamin D and 1,25-dihydroxyvitamin D by radioimmunoassay using radioiodinated tracers. Methods Enzymol.

[24] DiaSorin 25-Hydroxyvitamin D 125I RIA Kit: for the quantitative determination of the 25-OH-D and other hydroxylated metabolites in serum or plasma. Instruction Manual.

[25] Symmans WF, Peintinger F, Hatzis C, Rajan R, Kuerer H, Valero V (2007). Measurement of residual breast cancer burden to predict survival after neoadjuvant chemotherapy. J. Clin. Oncol.

[26] Spyratos F, Ferrero-Pous M, Trassard M, Hacene K, Phillips E, Tubiana-Hulin M (2002). Correlation between MIB-1 and other proliferation markers: clinical implications of the MIB-1 cutoff value. Cancer.

[27] Railo M, Lundin J, Haglund C, von Smitten K, von Boguslawsky K, Nordling S (1997). Ki-67, p53, Er-receptors, ploidy and S-phase as prognostic factors in T1 node negative breast cancer. Acta Oncol.

[28] Callagy GM, Pharoah PD, Pinder SE, Hsu FD, Nielsen TO, Ragaz J (2006). Bcl-2 is a prognostic marker in breast cancer independently of the Nottingham Prognostic Index. Clin. Cancer Res.

[29] Hudis CA, Barlow WE, Costantino JP, Gray RJ, Pritchard KI, Chapman JA (2007). Proposal for standardized definitions for efficacy end points in adjuvant breast cancer trials: the STEEP system. J. Clin. Oncol.

[30] Botella-Carretero JI, Alvarez-Blasco F, Villafruela JJ, Balsa JA, Vazquez C, Escobar-Morreale HF (2007). Vitamin D deficiency is associated with the metabolic syndrome in morbid obesity. Clin. Nutr.

[31] Clemens TL, Adams JS, Henderson SL, Holick MF (1982). Increased skin pigment reduces the capacity of skin to synthesise vitamin D3. Lancet.

[32] Holick MF (2006). Resurrection of vitamin D deficiency and rickets. J. Clin. Invest.

[33] Macdonald HM, Mavroeidi A, Barr RJ, Black AJ, Fraser WD, Reid DM (2008). Vitamin D status in postmenopausal women living at higher latitudes in the UK in relation to bone health, overweight, sunlight exposure and dietary vitamin D. Bone.

[34] Muscogiuri G, Sorice GP, Prioletta A, Policola C, Casa SD (2010). Association of vitamin D with insulin resistance and beta-cell dysfunction in subjects at risk for type 2 diabetes: comment to Kayaniyil et al. Diabetes Care.

[35] Thomas MK, Lloyd-Jones DM, Thadhani RI, Shaw AC, Deraska DJ, Kitch BT (1998). Hypovitaminosis D in medical inpatients. N. Engl. J. Med.

[36] Tseng M, Giri V, Bruner DW, Giovannucci E (2009). Prevalence and correlates of vitamin D status in African American men. BMC Public Health.

[37] Freedman LP (1999). Transcriptional targets of the vitamin D3 receptor-mediating cell cycle arrest and differentiation. J. Nutr.

[38] Jensen SS, Madsen MW, Lukas J, Binderup L, Bartek J (2001). Inhibitory effects of 1alpha,25-dihydroxyvitamin D(3) on the G(1)-S phase-controlling machinery. Mol. Endocrinol.

[39] Sinkkonen L, Malinen M, Saavalainen K, Vaisanen S, Carlberg C (2005). Regulation of the human cyclin C gene via multiple vitamin D3-responsive regions in its promoter. Nucleic Acids Res.

[40] Chaudhry M, Sundaram S, Gennings C, Carter H, Gewirtz DA (2001). The vitamin D3 analog, ILX-23-7553, enhances the response to adriamycin and irradiation in MCF-7 breast tumor cells. Cancer Chemother. Pharmacol.

[41] Abe-Hashimoto J, Kikuchi T, Matsumoto T, Nishii Y, Ogata E, Ikeda K (1993). Antitumor effect of 22-oxa-calcitriol, a noncalcemic analogue of calcitriol, in athymic mice implanted with human breast carcinoma and its synergism with tamoxifen. Cancer Res.

[42] Mohr SB, Gorham ED, Alcaraz JE, Kane CJ, Macera CA, Parsons JK (2011). Serum 25-hydroxyvitamin D and prevention of breast cancer: pooled analysis. Anticancer Res.

[43] Hatse S, Lambrechts D, Verstuyf A, Smeets A, Brouwers B, Vandorpe T (2012). Vitamin D status at breast cancer diagnosis: correlation with tumor characteristics, disease outcome, and genetic determinants of vitamin D insufficiency. Carcinogenesis.

